# “In Situ” Orbital Correlations

**DOI:** 10.1021/acs.accounts.5c00826

**Published:** 2026-06-12

**Authors:** Xuhui Lin, Huaiyu Zhang, Changwei Wang, Zexing Cao, Yirong Mo

**Affiliations:** † Hunan Key Laboratory of Super Microstructure and Ultrafast Process, School of Physics, 12570 Central South University, Changsha, Hunan 410083, China; ‡ Institute of Computational Quantum Chemistry, College of Chemistry and Material Science,Hebei Normal University, Shijiazhuang 050024, China; § Key Laboratory for Macromolecular Science of Shaanxi Province, School of Chemistry & Chemical Engineering, Shaanxi Normal University, Xi’an 710119, China; ∥ State Key Laboratory of Physical Chemistry of Solid Surfaces and Fujian Provincial Key Laboratory of Theoretical and Computational Chemistry, College of Chemistry and Chemical Engineering, 12466 Xiamen University, Xiamen 361005, China; ⊥ Department of Nanoscience, Joint School of Nanoscience and Nanoengineering, 14616 University of North Carolina at Greensboro, Greensboro, North Carolina 27401, United States

## Abstract

Orbital correlation diagrams are central to chemistry. Based on the symmetry compatibility and orbital overlap amplitude, they link the energy-ordered frontier molecular orbitals (MOs) of reactants and products and have long been a powerful and essential tool for understanding chemical interactions (reactions) and molecular properties. The frontier MOs typically include the highest occupied MOs (HOMOs) and the lowest unoccupied MOs (LUMOs), along with a few nearby orbitals of the reactants. However, it is also known that some reactions cannot be well explained with a few frontier MOs. The main drawback of traditional orbital correlation diagrams is that the orbital energies of the reactants shown in the diagram are calculated assuming they are in free, isolated states. But orbital energy levels can be significantly shifted by external fields and the existence of neighboring molecules. In other words, orbital energy levels can be notably reshuffled when we put reactants “physically” (via electrostatic interactions, Pauli repulsion, and van der Waals interactions) together, even without “chemical” interactions (via orbital mixtures or electron transfers).

Here, we introduce a novel concept, “in situ” orbital correlation, and demonstrate its applications. This concept is based on our developed block-localized wave function (BLW), which is the simplest variant of ab initio valence bond (VB) theory. The uniqueness of the BLW method lies in its ability to derive orbital energies of a molecule self-consistently in the presence of other species or external fields, as a BLW solution essentially corresponds to a hypothetical diabatic (or resonance) state, a mathematical construct in which all electron transfers between interacting species are “disabled”. In such a way, we can correlate orbitals by considering the field (physical) effects from neighboring species even without any orbital (chemical) interactions.

This “in situ” orbital correlation concept was first proposed in the study of the activation mechanism of CO by the diboryne compound B_2_(NHC^R^)_2_, where we demonstrated that when CO approaches B_2_(NHC^R^)_2_, there is a HOMO–LUMO swap in B_2_(NHC^R^)_2_ primarily due to the Pauli repulsion from the carbon lone pair of CO, leading to the compatibility of HOMO and HOMO-1 of B_2_(NHC^R^)_2_ with both π* orbitals of CO. Since then, this concept has been adopted in much of our research. For instance, in our most recent study of NCCL^–^ anions (L = N_2_, CO, CS), which exhibit notable geometric differences, “in situ” orbital correlation diagrams reveal an orbital swap in the fragment NCC^–^ with the approach of the ligand L and subsequently confirm the C(0) theory proposed by the Frenking group. Previously, we explored the “anti-electrostatic” nature of the Al–Mg bond and confirmed that the bond is purely ionic. This contradicts the view from frontier orbitals of Al­(I) and Mg compounds, which exhibit a perfect match for a dative covalent bond between them. Now, with the help of the “in situ” orbital correlation diagram, it becomes obvious that the metal–metal bond is a typical ionic bond, because when the Mg compound is brought close, the energy level of the HOMO of Al­(I) compound decreases significantly, leading to a reversal of the HOMO–LUMO energy level order and the extension of the HOMO–LUMO band gap and subsequently minimal probability of any electron transfer. We expect that the novel concept of “in situ” orbital correlation will fundamentally enrich our understanding of chemical reactions, electron transfer pathways, and molecular bonding.

## Graphical abstract

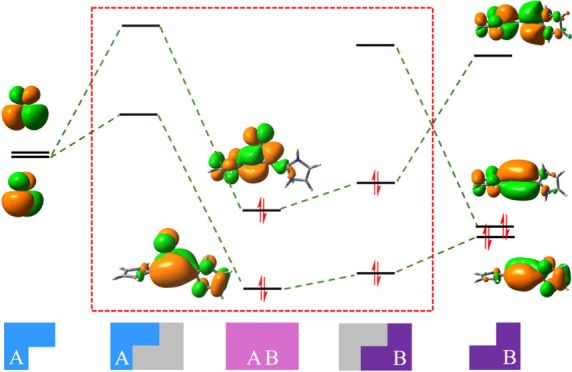

## Key References

-

Zhang, H. ; Cao, Z. ; Wu, W. ; Mo, Y. , The Transition-Metal-Like Behavior of B_2_(NHC)_2_ in the Activation of CO: HOMO–LUMO Swap Without Photoinduction Angew. Chem. Int. Ed. 2018, 57, 13076–13081.10.1002/anie.20180595230084124 ^1^ *In the study of the CO activation mechanism by diboryne, the HOMO–LUMO switch was observed in the diabatic state derived with our block-localized wave function (BLW) method, and the concept of “in situ” orbital correlation was first introduced.*
-

Ma, T. ; Wang, X. ; Peng, X. ; Li, J. ; Yin, S. ; Mo, Y. ; Wang, C. External electric fields drive the formation of P → C dative bonds. Chem. Sci. 2025, 16, 8542–8554 40242842 10.1039/d5sc01701gPMC11997864.^2^ *“In situ” orbital correlation diagrams illustrate the formation of dative bonds between PH*_3_ *and curved carbon-based nanostructures, driven by external electric fields (EFs) that serve as a catalytic force.*
-

Lin, X. ; Mo, Y. Chemical Bonding from the Perspective of In Situ Orbital Correlation. J. Phys. Chem. Lett. 2025, 16, 10811–10815 41065026 10.1021/acs.jpclett.5c02704PMC12536439.^3^ *Traditional orbital correlation suggests a dative (covalent) bonding nature in lithium–aluminum dimetallocenes, but “in situ” orbital correlation diagrams correctly show that the Li–Al bond is purely ionic.*
-

Wei, J. ; Ma, R. ; Song, J. ; Duan, Y. ; Li, X. ; Zhang, H. ; Mo, Y. Distinct Difference in the Geometries of NCCL^–^ Anions (L = N_2_, CO, CS): A Balance Between π Conjugation and Steric Repulsion. Inorg. Chem. 2025, 64, 22210–22218 41157990 10.1021/acs.inorgchem.5c04439PMC12606702.^4^ *“In situ” orbital correlation diagrams reveal an orbital switch in the fragment NCC with the approach of the ligand L, and confirm the carbone C(0) theory proposed by the Frenking group*.

## 1 Introduction

Orbital correlation diagrams are a key tool in chemistry for understanding molecular structures and reaction mechanisms. They originate in molecular orbital (MO) theory, following Mulliken’s introduction of the concept of orbitals in 1932.^5^ Mulliken’s pioneering work on the electronic structure of molecules eventually laid the groundwork for correlating orbital energies with molecular properties such as bond angles.^6^ Subsequently, Walsh extended Mulliken’s idea and created “Walsh diagrams”, which exhibit the evolution of orbital energies along a particular geometric change, and proposed Walsh’s rules, which predict the shape of a small molecule from the behavior of frontier orbitals, including the highest occupied MO (HOMO) and the lowest unoccupied MO (LUMO).^7^ While Walsh’s diagrams deal with individual molecules, a major leap in the application of orbital correlation diagrams came from the study of reaction mechanisms involving multiple molecules. Woodward and Hoffmann constructed orbital correlation diagrams for pericyclic reactions to match the symmetry of reactant orbitals with the symmetry of product orbitals along a proposed reaction pathway.^8^ According to the Woodward–Hoffmann rules, reactions will be “symmetry-allowed” if bonding reactant orbitals transform into bonding product orbitals without encountering a high-energy, symmetry-imposed barrier.

The orbital correlation diagrams currently in widespread use, however, are primarily based on frontier MOs.^9^ It is generally assumed that reactions can be well elucidated based on a few frontier MOs and a few nearby orbitals of reactants and products. Orbital correlation diagrams connect the energy-ordered orbitals of reactants and products based on the compatibility of symmetries or, more generally, on the amplitude of orbital overlaps, and have become an illuminating and powerful tool for understanding chemical reactions and molecular properties.^10,11^ The order of these orbital energy levels is critical in the estimation of the magnitude of interactions, though it is largely qualitative or semiquantitative. While the HOMO–LUMO gap is widely used as a measure of the kinetic stability and chemical reactivity of molecular systems,^12^ we caution that orbital interaction depends not only on the energy gap but also on the magnitude of the overlap and the coupling integral between the interacting orbitals. As an exemplary case, Figure 1a shows the formation of the dative bond between ammonia (NH_3_) and borane (BH_3_) based on the HOMO of NH_3_ and LUMO of BH_3_, which correctly highlights the electron transfer from NH_3_ to BH_3_ in the formation of the N–B dative (covalent) bond and the origin of the stability of the complex. However, it is also known that many reactions cannot be explained simply in terms of frontier MOs.^13^ For instance, the degenerate HOMOs of B^–^ (triplet) have higher energy levels than the LUMOs of Ca, suggesting a rapid electron transfer pathway from B^–^ to Ca. But detailed analyses of the triplet CaB^–^ molecule showed that the electrons move in the opposite direction, from Ca to B^–^.^14^ Recently, we studied the antielectrostatic and purely ionic nature of the main group metal–metal bonds between a low valent aluminum compound [(BDI)­Al], which is a stable aluminum analogue of a carbene with a lone pair on Al, and a set of cationic BDI alkaline earth (Ae) metal complexes [(BDI)­Ae]^+^ (BDI ligand refers to the ligand HC­(CMeNAr)_2_^–^ (Ar = 2,6-*i*Pr_2_C_6_H_3_)).^15–18^ Based on the frontier orbitals (Figure 1b), we identified the perfect match between the HOMO of [(BDI)­Al] and the LUMO of [(BDI)­Ae]^+^ in terms of both symmetry and energy compatibilities. Consequently, the metal–metal bond was expected to be dative, much like the B–N bond in the H_3_N-BH_3_ complex. However, further analyses revealed that the metal–metal bonds are typical ionic bonds in nature and can be well interpreted in purely Coulombic terms, including electrostatics and polarization.^19^ The conclusion of ionic bonding nature is also supported by other computational analyses, such as the quantum chemical topological method (QTAIM).

**Figure 1:**
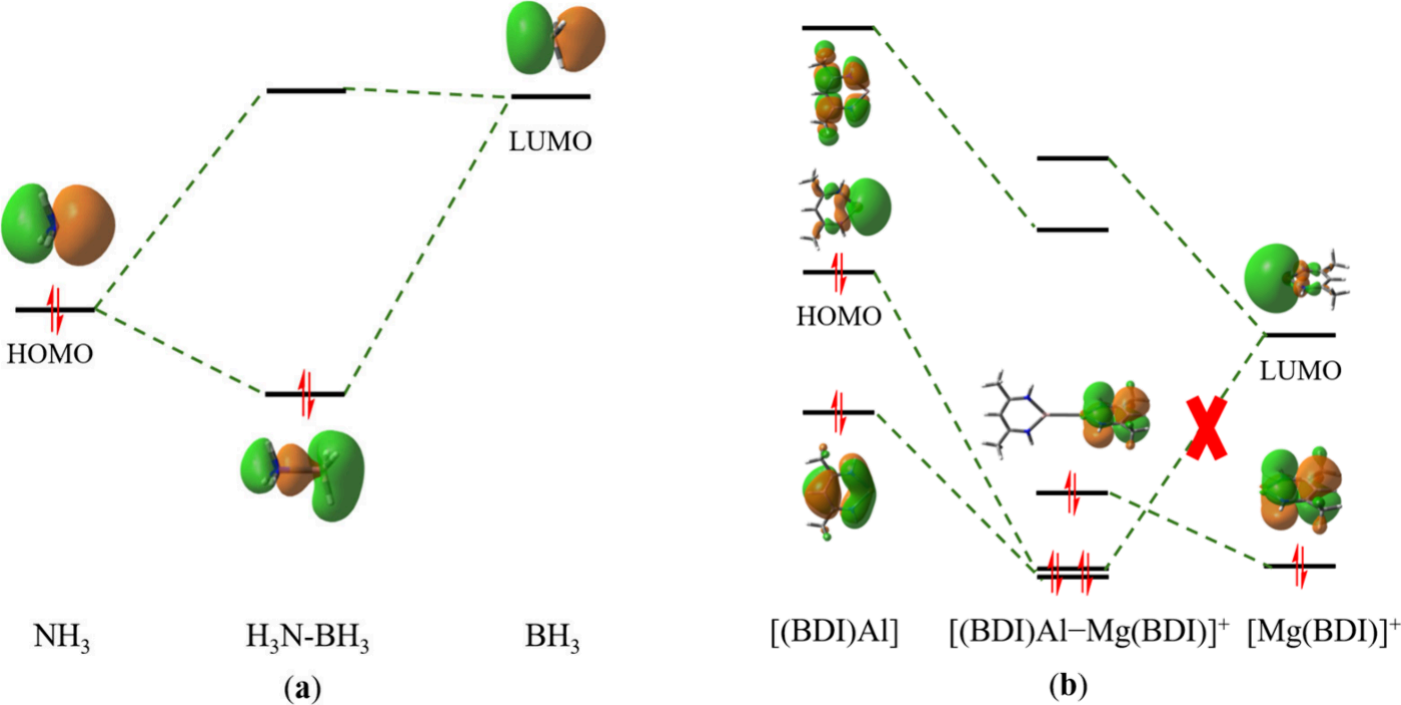
Traditional orbital correlation diagrams: (a) dative bond from the HOMO of NH_3_ to the LUMO of BH_3_; (b) ionic metal–metal bond, which is inconsistent with the frontier orbital correlations.

We believe that the main drawback of current orbital correlations lies in the orbital energy levels of reactants, which are derived from their states of separation and isolation. Obviously, orbital energies will be influenced by external fields and the existence of neighboring molecules and thus will be notably shifted when we put reactants “physically” together, even without any “chemical” (i.e., orbital) interactions. But the question is how to define such a hypothetical resonance state theoretically. This is equivalent to the proper definition of the “diabatic” noncharge transferred state in the generalized Mulliken-Hush two-state approximation in the study of electron transfers.^20–22^ In electron transfer theory, diabatic states are chemically intuitive, charge-localized states where the electron is purely on the “donor” or “acceptor”. While adiabatic states are eigenstates of the full electronic Hamiltonian, diabatic states are not, and their constructions are thus nontrivial.^23^ Common approaches include fragment-charge-difference methods, Boys localization, generalized Mulliken–Hush, block diagonalization, and constrained DFT, each of which enforces charge localization on the donor or acceptor to define the diabatic surfaces and extract the coupling. The BLW method, however, provides an optimal solution for constructing diabatic states.

## 2 Methodology and the Concept of “In Situ” Orbital Correlation

While a diabatic state wave function cannot be derived from MO and DFT-based methods directly without introducing any significant approximations, it can be well-defined and self-consistently optimized from modern ab initio valence bond (VB) theory in the form of Heitler–London–Slater–Pauling (HLSP) function.^24–29^ An HLSP function corresponds to a resonance (diabatic) structure with *n* chemical bonds and can be expanded to 2^*n*^ Slater determinants. Different from MO theory, orbitals in VB theory are generally nonorthogonal, and this complicates the computations of ab initio VB theory. The high computational requirements limit the widespread applications of ab initio VB methods. One way to simplify an HLSP and achieve the efficiency of MO or DFT methods is to express each chemical bond with a doubly occupied bond orbital. In this way, an HLSP can be conveniently defined using a single Slater determinant. Following this line of thought, we proposed and developed the block-localized wave function (BLW) method, which defines a diabatic state with a single Slater determinant and thus is the simplest variant of ab initio VB theory with the computational efficiency of MO or DFT.^30–33^ The general idea of the BLW method is the partition of a whole system into several blocks, and electrons are allowed to delocalize only within one block. In other words, all orbitals are block-localized. Orbitals within the same block are constrained to be orthogonal, like the MO method. But orbitals from different blocks are nonorthogonal, like the VB method.

The unique feature of the BLW method is that it is an ab initio method that can quantitatively and self-consistently derive the wave function of a diabatic state, in which all orbital mixing or interactions are suppressed. In other words, it can address the impact of electron transfer on molecular geometry, energetics, and spectral properties. Initially, the BLW method was developed at the HF level, but if we keep all the equations unchanged and simply replace the Fock matrix with the DFT matrix, it is easy to implement the BLW method into the DFT algorithm.^32^ For the example of two interacting species A and B, which concern orbital correlation diagrams, we can define the wave function Ψ^BLW^ for the intermediate diabatic state as

1 $$\Psi^{\mathrm{BLW}}=Â\left(\Phi_{A}\Phi_{B}\right)$$

where all orbitals are block-localized to either A or B, and the orbital energies of A and B can be subsequently derived. Compared to their separate states, the orbital energy levels are shifted due to the perturbation by the electric field and Pauli repulsion (i.e., steric effect as a whole) generated by the other species (and external fields if they are incorporated in the study). The self-consistent solution of eq 1 can be accomplished using successive Jacobi rotation^30^ or Gianinettia et al.’s algorithm.^34,35^ The latter generates the pseudo-Hartree–Fock–Roothaan equation for each block

2 $$F_{i}^{\prime }C_{i}=S_{i}^{\prime }C_{i}\varepsilon_{i}$$

where ***F***_*i*_^′^ and ***S***_*i*_^′^ (*i* = A or B) are effective Fock and overlap matrices with the constraint of ***C***_*i*_^+^ ***S***_*i*_^′^ ***C***_*i*_ = 1. Since all orbitals are expanded within A or within B, there are no orbital extensions between A and B, and thus, there is no electron transfer. However, electrostatic interaction, Pauli repulsion, and polarization effect between A and B are retained. Once we have the optimal block-localized orbitals ***C***_*i*_ derived, we can obtain the orbital energies as ***ε***_*i*_ is a diagonal matrix.

Based on the energy levels derived from eq 2, we can demonstrate the evolution of orbital energies from a free optimal state to a free distorted state for A or B, then to a diabatic state and finally to an adiabatic state involving both A and B. Notably, the correlations from diabatic states to the final adiabatic state are defined as “in situ” orbital correlations that are expected to provide more objective insights into chemical bonding and reaction than traditional orbital correlation diagrams. It should also be noted that we are not changing traditional orbital correlation diagrams. Rather, we are adding intermediate states between reactants and products to the traditional orbital correlation diagrams. In this way, multilayered information can fundamentally enrich our understanding of chemistry. In the following section, we will present a few examples. All computations were performed using the in-house version of GAMESS software,^36^ to which our BLW method is ported.

## 3 Demonstrations of the Concept of “In Situ” Orbital Correlation

### 3.1 Dative Bond in Ammonia Borane

For the H_3_N–BH_3_ example shown in Figure 1a, we computed the orbital energy levels of both NH_3_ and BH_3_ at their complex geometry, with orbital interactions disabled, using the BLW method. It is known that the orbital interaction or electron transfer stabilizes the complex considerably.^37^ Compared with Figure 1a, Figure 2 shows an updated “in-situ” orbital correlation diagram. When NH_3_ and BH_3_ are put together (with subscript sign “c” meaning constrained) from their free states (with subscript “f”), the HOMO of NH_3_ lowers the energy, but the HOMO-1 of BH_3_, which is symmetrically compatible with the HOMO of NH_3_, raises the energy. In general, electrostatic interactions, Pauli repulsion, and polarization effects all contribute to orbital level shifts. In the case of H_3_N–BH_3_, these shifts dramatically reduce the energy gap between both occupied MOs from 6.90 eV in their free states to only 0.45 eV in their diabatic state. The subsequent chemical interaction (orbital mixing) between the two orbitals leads to one being at a high energy level and the other at a low energy level. The one with a high energy level further interacts with the LUMO of BH_3_ and gets stabilized. Obviously, the “in situ” orbital correlation diagram framed in red dashed lines in Figure 2 provides additional information on orbital interactions compared with Figure 1, thus deepening our understanding of the bonding details.

**Figure 2:**
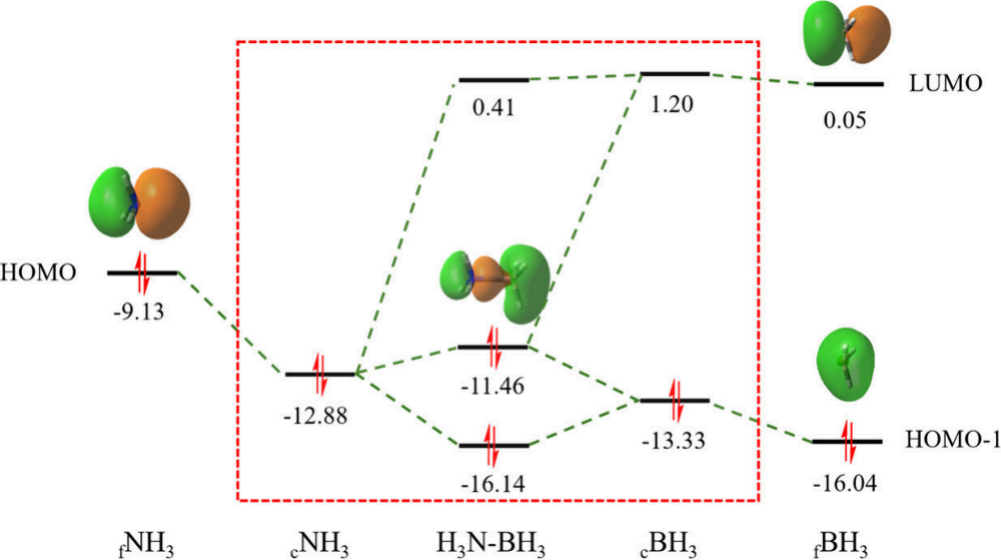
“In situ” orbital correlation diagram shown for the formation of NH_3_BH_3_ in the red frame. _f_X and _c_X mean a free X and a constrained X in the complex BLW state, respectively (X = NH_3_ and BH_3_). Orbital energy levels (in eV) are based on the M06-2*X*/6–311+G­(d,p) computations.

### 3.2 Diels–Alder Reaction

One of the most important reactions in chemistry is the Diels–Alder reaction between a conjugated diene and a substituted alkene termed a dienophile, to form a substituted cyclohexene derivative. Here, we exemplified the interaction between butadiene and ethylene and explored the evolution of orbital energy levels in the formation of the transition state (TS). Figure 3 shows the “in situ” orbital correlations for the Diels–Alder reaction. With the approach between diene and dienophile, all occupied orbitals raise their energy levels, notably the HOMO-1 of diene and the HOMO of dienophile, due to electrostatic and Pauli repulsions. The consequence is the reduction of HOMO–LUMO gaps in both directions. The gap from diene to dienophile is 8.03 eV, compared with 8.24 eV from dienophile to diene. The mutual electron transfers make the overall net electron movements insignificant, though the energy difference between the BLW state and the adiabatic state, which is the charge transfer energy component for the binding between diene and dienophile, reaches 38.6 kcal/mol after the basis set superposition error (BSSE) correction (0.6 kcal/mol). This energy term is derived from the energy decomposition (BLW-ED) analysis based on the BLW method.^31^ Figure 4 shows the electron density difference (EDD) map, derived from the electron densities of both the adiabatic state (regular DFT) and the diabatic state (BLW, with electron transfers between diene and dienophile quenched). The EDD map reveals electron accumulation in the shared bonding region between the terminal carbon atoms of the diene and the dienophile. The shared bonding areas eventually result in two new C–C bonds.

**Figure 3:**
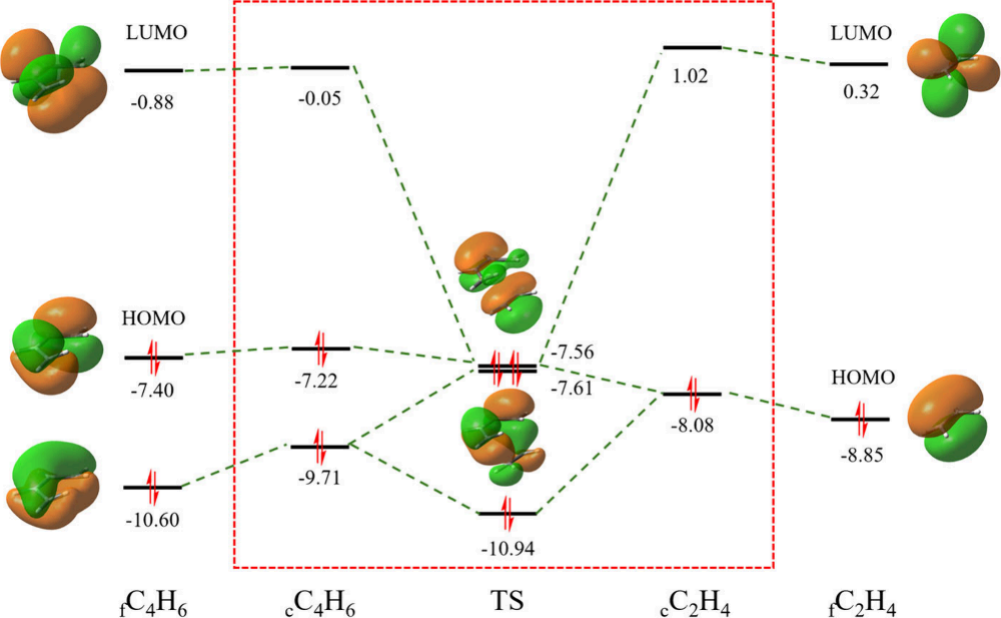
“In situ” orbital correlation diagram for the Diels–Alder reaction shown in the frame. _f_X and _c_X mean a free X and X in the complex BLW state, respectively (X = C_4_H_6_ and C_2_H_4_). TS is the transition state. Orbital energy levels (in eV) are based on the M06–2*X*/6–311+G­(d,p) computations.

**Figure 4:**
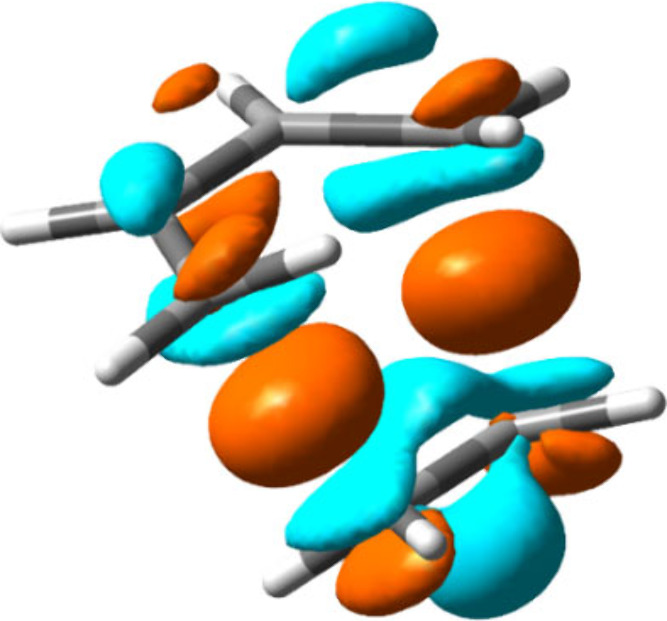
Electron density difference (EDD) map for the TS structure in the Diels–Alder reaction. The orange and cyan colors denote the increase and decrease of electron densities, respectively (with isovalue 0.003 e Å^–3^).

### 3.3 Metal–Metal Ionic Bonds

For the two cases above, although “in situ” orbital correlations enrich our understanding of bonding and reaction mechanisms in greater detail, traditional correlation diagrams still work well, at least qualitatively. In some situations, however, traditional correlation diagrams will simply fail, as we observed in Figure 1b, where the HOMO of [(BDI)­Al­(I)] is of higher energy than the LUMO of [(BDI)­Ae]^+^, e.g., by 1.62 eV for Ae = Mg, and both orbitals are symmetrically compatible. Yet, BLW-ED analyses showed that, unlike H_3_N-BH_3_, the charge transfer energies in Al–Re (Re = Ca and Mg) compounds are negligible (7.5 and 3.2 kcal/mol, respectively) compared with the overall binding energies (66.4 and 45.2 kcal/mol, respectively), and further BLW geometry optimizations of these complexes with the Al lone pair strictly localized on itself (thus no dative covalency at all) resulted in similar bonding distances and strengths.^19^ In other words, no dative bond can be forged between Al­(I) and Re.

In retrospect of these typical ionic metal–metal bonds now, we plotted the correlation diagram with the example of the Al–Mg compound with the orbital energy levels derived from the diabatic state (Figure 5). With the “in situ” orbital correlation diagram, we can observe that the HOMO of [(BDI)­Al­(I)] is remarkably stabilized by the cationic [(BDI)­Mg]^+^ when they get close by as much as 6.34 eV, leading to a HOMO–LUMO gap (8.03 eV) with the HOMO in lower energy than the LUMO of [(BDI)­Mg]^+^. The reverse of the HOMO and LUMO order and the expanded HOMO–LUMO gap result in negligible orbital interaction. Interestingly, the gap between the LUMO of [(BDI)­Al­(I)] and the HOMO of [(BDI)­Mg]^+^ is also reduced remarkably, but their orbital overlap is close to zero due to the incompatibility of orbital symmetries, and thus any orbital interaction is similarly unlikely. As there are no favorable HOMO–LUMO interactions in either direction, all occupied orbitals maintain their energy levels during complexation (changes are less than 0.08 eV). There is essentially no electron transfer between [(BDI)­Al­(I)] and [(BDI)­Mg]^+^, confirming the perfect ionicity of the Al–Mg bond. Obviously, the electrostatic attraction from [(BDI)­Mg]^+^ to the lone pair of Al­(I) is the driving force for the dramatic decrease of the HOMO of [(BDI)­Al­(I)] and the stability of the ionic Al–Mg bond.

**Figure 5:**
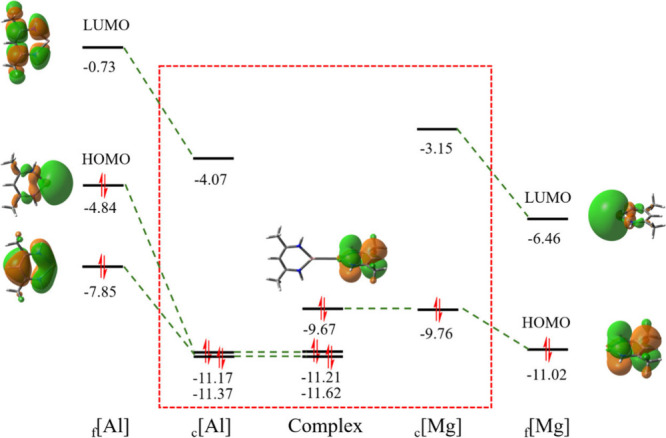
“In situ” orbital correlation diagram showing the Al–Mg ionic bond between [(BDI)­Al­(I)] and [(BDI)­Mg]^+^. _f_X and _c_X mean a free X and X in the complex BLW state, respectively (X = [(BDI)­Al­(I)] and [(BDI)­Mg]^+^). “Complex” refers to [(BDI)­Al–Mg­(BDI)]^+^. Orbital energy levels (in eV) are based on the M06-2*X*/6-311+G­(d,p) computations.

The recently synthesized lithium–aluminum heterobimetallic dimetallocene, i.e., ^5^CpAl–Li^5^Cp (^5^Cp = isopropyl-substituted cyclopentadienyl ligand), exhibits similar features, though both moieties are neutral in this case.^38^ While it was expected that the Al–Li bond contains a considerable covalent component from the lone pair of Al to the cationic Li (see HOMO of [Al] and LUMO of [Li] in Figure 6), theoretical analyses showed that the Al–Li bond exhibits high ionic character, and the bond is further stabilized by attractive dispersion interactions between the bulky isopropyl groups. Similar bonding patterns have been observed in other neutral dimetallocene derivatives, including CpAl–LiCp and *CpAl–Li*Cp, though there is much less dispersion stabilization due to the relatively small substituents in these analogs.

**Figure 6:**
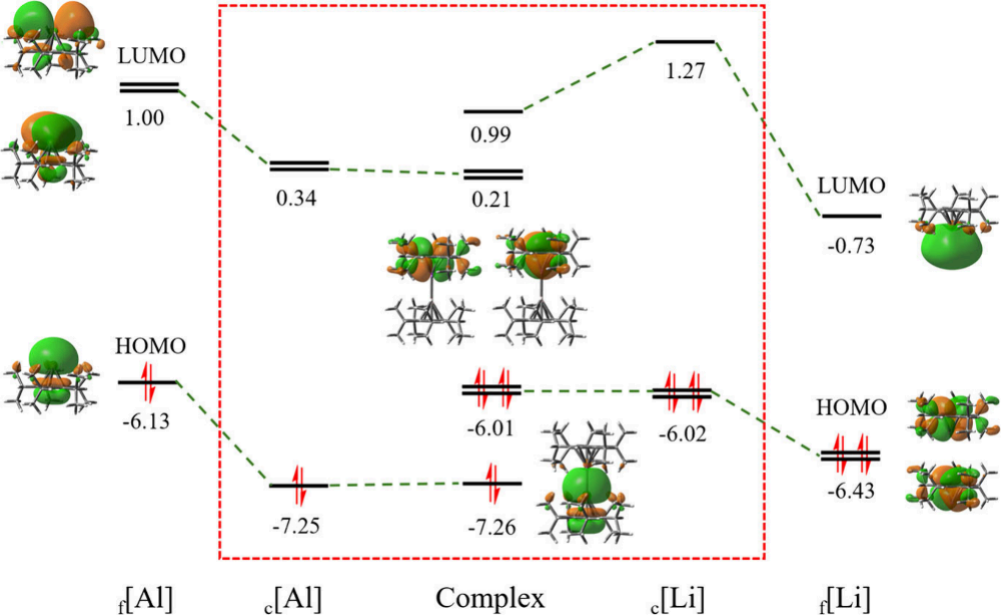
“In-situ” orbital correlation diagram showing the Al–Li ionic bond in ^5^CpAl–Li^5^Cp. _f_X and _c_X mean a free X and X in the complex BLW state, respectively (X = ^5^CpAl and Li^5^Cp). “Complex” refers to ^5^CpAl–Li^5^Cp. Orbital energy levels (in eV) are based on the M06–2X/Def2-TZVPP computations.

Since traditional orbital correlation diagrams are unable to capture the ionic character of the Al–Li bonds in these compounds, we constructed the “in situ” orbital correlation diagrams as shown in Figure 6 for ^5^CpAl–Li^5^Cp.^3^ Figure 6 shows that the approach of ^5^CpLi to ^5^CpAl stabilizes the latter’s HOMO considerably (by 1.12 eV), and in the meantime increases the former’s LUMO even more (by 2.00 eV). As a consequence, the HOMO–LUMO gap between the donor ^5^CpAl and the acceptor ^5^CpLi increases substantially, indicating a drastically reduced orbital interaction. Similar to the complex of [(BDI)­Al­(I)] and [(BDI)­Mg]^+^ (Figure 5), the energy gap between the degenerate LUMOs of ^5^CpAl and the HOMOs of CpLi decreases when the two species are put together. But their symmetries are incompatible, so no orbital interaction occurs (with changes in HOMO energy levels of less than 0.01 eV). Therefore, the “in situ” orbital correlation diagram provides insight into the Al–Li bond and confirms that it is purely ionic, stabilized primarily by electrostatic and polarization interactions, as well as dispersion.

### 3.4 Activation of CO by Diboryne

The importance of the concept of “in situ” orbital correlation is best illustrated by the example of CO activation by main-group compounds, which lack d orbitals, unlike transition metals. Currently, there is a trend toward developing catalysts based on Earth-abundant and non-noble metals or main-group elements for the activation of small molecules such as CO, as this would be an economical and environmentally benign process. The activation of CO is traditionally interpreted as electron back-donation from the metal center to CO in the form of d→π*. Unlike transition metals, main group elements usually do not have d valence orbitals. Yet, main-group metallomimetics have been envisioned as another attractive option, and there is a growing number of main-group compounds that are usually multibonded and unsaturated and behave similarly to transition metal complexes.^39,40^ Of particular interest and promise are boron-based molecules, which can activate small molecules and cleave stable covalent bonds such as C–H.^41^ Significantly, the Braunschweig group synthesized a diboryne (B≡B) stabilized by N-heterocyclic carbenes (B_2_(NHC^R^)_2_) and demonstrated that this compound can bind and activate CO.^42,43^ Many follow-up computational studies of this diboryne and its reaction mechanism with CO have been conducted. However, these computational studies are largely based on conventional molecular orbital or density functional theory, which assumes that all electrons are delocalized over the whole molecular system. To better understand the interactions between CO and diboryne, it is preferred to start with localized orbitals (i.e., diabatic state). While many post-SCF techniques have been proposed to derive localized orbitals from delocalized molecular orbitals, these localized orbitals are not optimal, and their corresponding energy levels are thus pushed up. In this regard, alternative theoretical methods are needed to reveal hidden information.

We subsequently employed the BLW method to probe how triply bonded diboryne activates CO and couples CO molecules by defining diboryne and CO as two blocks in BLW computations.^1^ Figure 7 shows the evolutions and “in situ” interactions of orbitals. As expected, when CO and B_2_(NHC)_2_ are far away from each other with their slightly deformed geometries in the complex, the degenerate LUMOs of CO and the equally degenerate HOMOs of B_2_(NHC)_2_ have an energy gap of around 2.29 eV. Comparing the symmetries of these orbitals, however, we can see that only the HOMO π_1_ of diboryne is compatible with one of the LUMOs (π_∥_^*^) of CO, and the other HOMO π_2_ of diboryne and the other LUMO π_⊥_^*^ of CO are not symmetry-compatible and thus unable to interact with each other as their overlap is close to zero. When we put two species together in their complex form without any orbital interactions (i.e., a diabatic state), interestingly, we observed the reshuffling of orbital energy levels. While both initially degenerate antibond orbitals (π_∥_^*^ and π_⊥_^*^) of CO are pushed up and split, one of the HOMOs (π_2_) of diboryne is pushed up so much that it becomes a virtual orbital. In the meantime, another initially virtual orbital stabilizes, lowers its energy level, and becomes the HOMO. This type of HOMO–LUMO switch typically occurs via photoinduction. But here, the cause is primarily the Pauli repulsion, since the HOMO of CO is occupied by a lone pair of carbon. When CO approaches a diboryne, the head-to-head overlap between the HOMOs of the two species results in strong Pauli repulsion, pushing one orbital up. We note that traditional orbital correlation diagrams fail to capture such interesting phenomena; here, Pauli repulsion is essentially a physical effect, as the lone-pair orbital on the carbon of CO does not mix (interact) with the π_2_ orbital of diboryne. In traditional orbital correlation diagrams illustrating the CO activation mechanism, the carbon lone-pair orbital is omitted because it does not directly contribute to CO activation.

**Figure 7:**
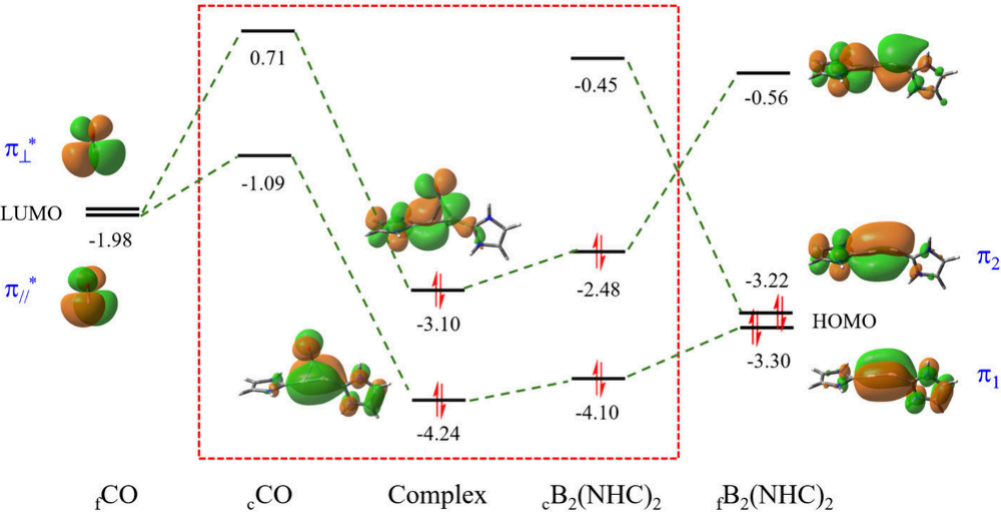
“In situ” orbital correlation diagram showing the activation of CO by diboryne. _f_X and _c_X mean a free X and X in the complex BLW state, respectively (X = CO and B_2_(NHC)_2_). “Complex” refers to the CO binding state CO-B_2_(NHC)_2_. Orbital energy levels (in eV) are based on the M06–2*X*/6–311+G­(d,p) computations.

Figure 7 shows that the “in situ” HOMO and HOMO-1 of diboryne are now symmetrically compatible with the LUMOs of CO, respectively. In other words, both the π antibonding orbitals of CO can accept electrons from diboryne. The significant electron back-donation weakens the CO triple bond and facilitates CO activation.

### 3.5 Proof of the Carbone theory

There is current interest in ketenyl anions/ynolates, which are readily accessible precursors that can be converted into ketene derivatives under milder conditions.^44^ Gessner and Frenking et al. isolated a few valence-isoelectronic compounds of NCCL^–^, including the cyanodiazomethanide anion (NCCNN^–^) and cyanothioketenyl anion (NCCCS^–^), and interpreted their structures in terms of NCC^–^←L σ-donation and in-plane and out-plane NCC^–^→L π-backdonation interactions within the carbon(0) or carbones theory.^45–47^ Traditionally, carbon is tetravalent with four bonds. But in carbon(0) compounds that have the general form CL_2_, the central carbon atom retains two unbonded lone pairs of electrons and forms two dative bonds as the electron acceptor from two ligands L. Thus, the central carbon atom has a formal oxidation state of zero. In the linear NCC^–^, however, the lone electron pair on the terminal carbon atom moves outward along the main axis of the molecule. When a Lewis base approaches linearly, the carbon does not have a compatible vacant orbital to accept electrons with the σ symmetry, unless the lone pair is excited to another virtual orbital of π symmetry. Indeed, our recent BLW studies of a series of NCCL^–^ anions (L = N_2_, CO, CS) exhibit such orbital switches similar to the one shown in Figure 7.^4^ Figure 8 exemplifies the “in situ” orbital correlations in NCCCO^–^ (i.e., L = CO). Due to the strong Pauli repulsion from the lone pair of C in CO, the σ orbital of NCC^–^ is pushed up and the two electrons subsequently move to a virtual π orbital. The resulting virtual σ orbital is largely located on C of NCC^–^, and can accept the electron donation from the lone pair of C in CO. Thus, the “in situ” orbital correlation diagram confirms the carbone theory by the Frenking group.

**Figure 8:**
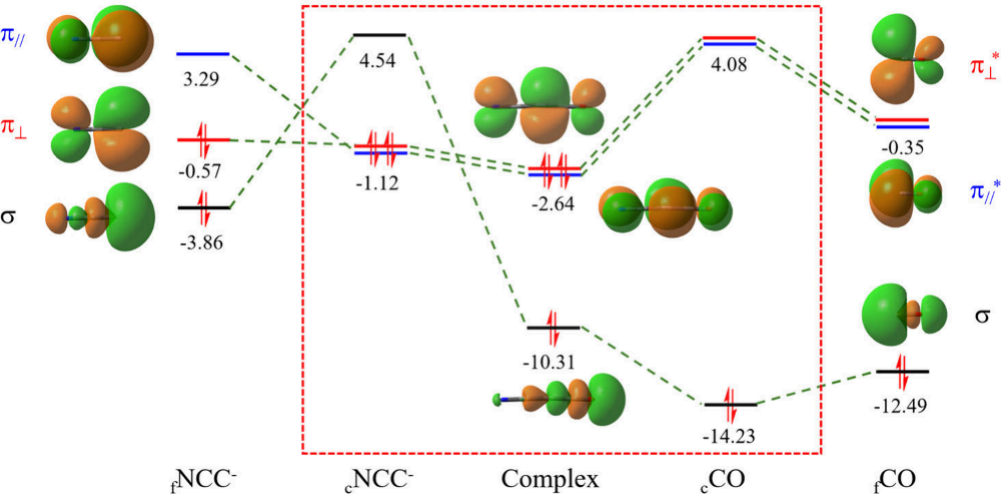
“In situ” orbital correlation diagram showing the bonding between NCC^–^ and CO. _f_X and _c_X mean a free X and X in the complex BLW state, respectively (X = NCC^–^ and CO). “Complex” refers to the anion NCCCO^–^. Orbital energy levels (in eV) are based on the M06-2*X*/6-311+G­(d) computations.

## 4 Final Note

Orbital correlation diagrams have become an important visual tool for interpreting and understanding chemical reaction mechanisms. They usually involve a few frontier MOs and exhibit their mixtures and energy-level changes from their separate, free states to the complex (product) state. However, such correlations may be oversimplified, ignoring key intermediate changes in the process. This is similar to elucidating the bonding nature based on bonding strength without exploring the detailed contributions to the bonding strength, because a weak bonding strength (e.g., weak CO_2_ absorption) may result from a balance between strong bonding and high deformation costs (e.g., CO_2_ bending). Fortunately, for the latter, numerous energy decomposition approaches (EDAs) have been developed and employed broadly. For orbital correlation diagrams, we believe that similar multilayered information can be inserted between the initial separate, free states and the final complex state.

Considering the influence of external physical fields on orbital energy levels, “in situ” orbital correlations capture the true orbital interactions and thereby reveal authentic electron-transfer pathways. Traditional orbital correlations start from the orbital energy levels derived from free and isolated species, but “in situ” orbital correlations are based on the energy levels derived from a hypothetical diabatic state where species are “physically” put together but without “chemical” interactions. Such a diabatic state is represented with a block-localized wave function (BLW), which is self-consistently optimized at the DFT level. In this way, we describe the orbital correlations progressively, namely from the initial free, separate states to the intermediate states and finally to the complex state. Our research shows that electrostatic interactions and Pauli repulsion often lead to orbital-level rearrangements, such as HOMO–LUMO swaps, that cause traditional orbital correlation diagrams to fail. Adding a new layer of information to traditional orbital correlation diagrams can elucidate the causes of orbital-level rearrangements. Similar to EDAs, we can define additional states between the initial and intermediate states and study orbital energy changes arising from electrostatics, Pauli repulsion, and polarization stepwisely. Subsequently, we can track the evolution of orbital energies due to electrostatics, Pauli repulsion, polarization, and charge transfer interactions. The exploration of detailed orbital energy changes and correlations can considerably enrich our understanding of chemistry. We expect that the novel concept of “in situ” orbital correlation will provide illuminating descriptions of chemical reaction mechanisms, molecular bonding, and electron-transfer pathways.
